# Supplementary material for: A Modular Bacteriophage T4 Nanoparticle Platform Enables Rapid Design of Dual COVID‐19‐Flu Mucosal Vaccines
Source: Small Sci. 2025 Jan 28;5(4):2400580. doi: 10.1002/smsc.202400580 (PMC12245071; doi:10.1002/smsc.202400580)
Supplement: Supplementary file 1 — Supplementary Material [file SMSC-5-2400580-s001.pdf]

Supporting Information

A Modular Bacteriophage T4 Nanoparticle Platform Enables Rapid Design of Dual COVID-19-Flu Mucosal Vaccines

Jingen Zhu[1*], Jian Sha[2,3,4], Himanshu Batra[1], Swati Jain[1], Xiaorong Wu[1], Emily K. Hendrix[2], Paul B. Kilgore[2], Keer Sun[2], Kenneth S. Plante[2,3,4,5], Jessica A. Plante[2,3,4,5], Jordyn Walker[2,5], Pan Tao[8], Ashok K. Chopra[2,3,4,6,7*], and Venigalla B. Rao[1*]

[1]Bacteriophage Medical Research Center, Department of Biology, The Catholic University of America, Washington, DC, 20064, USA.

[2]Department of Microbiology and Immunology, [3]Galveston National Laboratory, [4]Institute for Human Infections and Immunity, [5]World Reference Center for Emerging Viruses and Arboviruses, [6]Center for Biodefense and Emerging Infectious Diseases, [7]Sealy Institute for Vaccine Sciences, University of Texas Medical Branch, Galveston, TX, 77555, USA.

[8]State Key Laboratory of Agricultural Microbiology, College of Veterinary Medicine, Huazhong Agricultural University, Wuhan, Hubei, China

*Correspondence: zhuj@cua.edu; achopra@utmb.edu; rao@cua.edu

Supplemental Figures and Tables

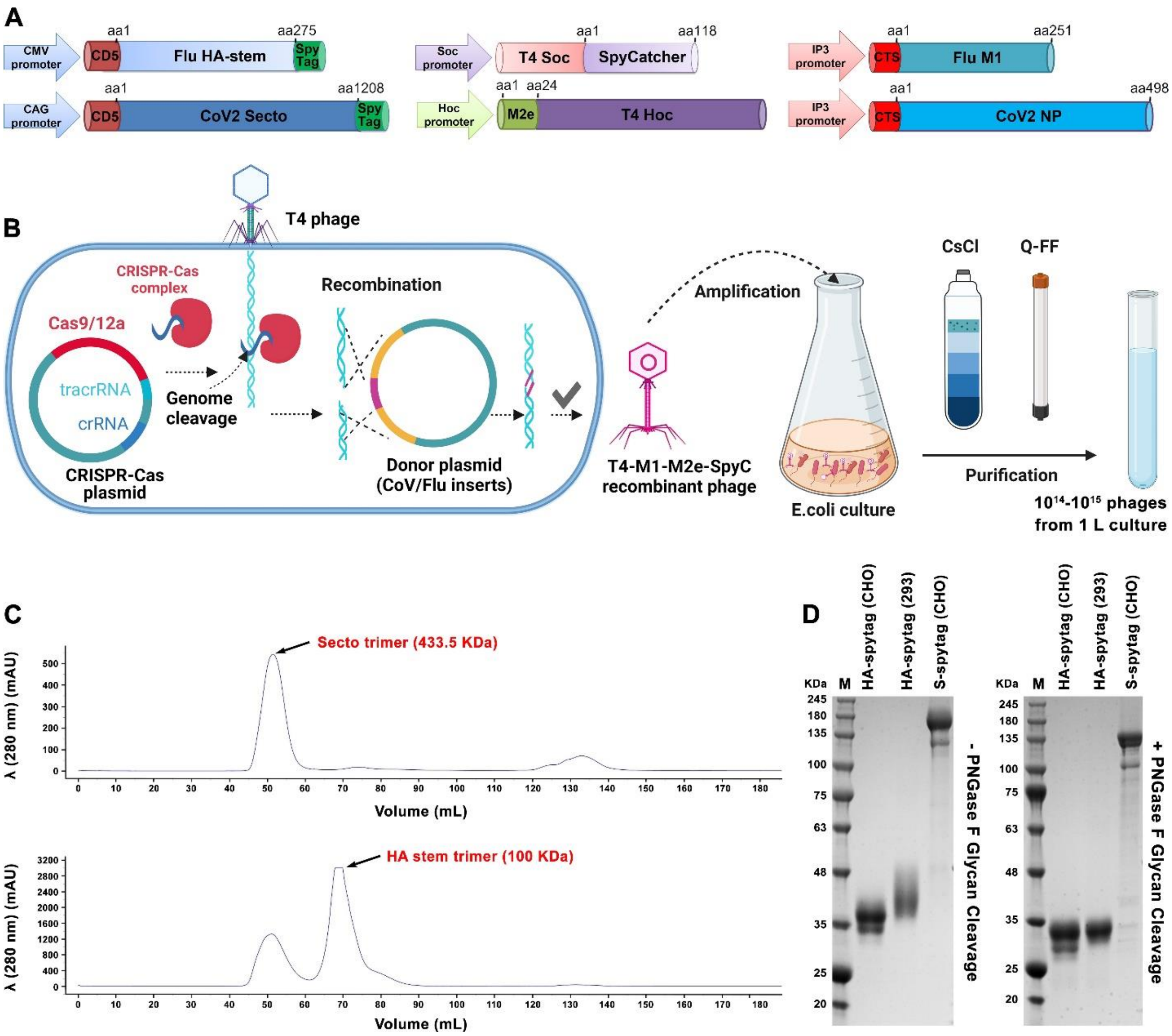

Figure S1. Components of the T4-CoV-Flu nanovaccine. A) Schematic of various gene expression cassettes for T4 *in vitro* display, *in vivo* display, and encapsidation. B) Schematic of CRISPR engineering scheme for the production of recombinant T4-M1-M2e-SpyCatcher phage. c and d Purification and characterization of S-ecto-Spytag trimers from ExpiCHO cells and HA stem-Spytag trimers from HEK293F cells. C) Size-exclusion chromatography (SEC) elution profiles of S-ecto-Spytag trimers (top) and HA stem trimers (bottom). D) SDS-PAGE patterns of SEC-purified HA stem-Spytag trimers (from ExpiCHO or HEK293F cells) and S-ecto-Spytag trimers (from ExpiCHO), both without (left) and with (right) the treatment of PNGase F glycan cleavage enzyme. Note the band shift of the envelope proteins following deglycosylation. The molecular weight standards (M) in KDa are shown on the left of each gel.

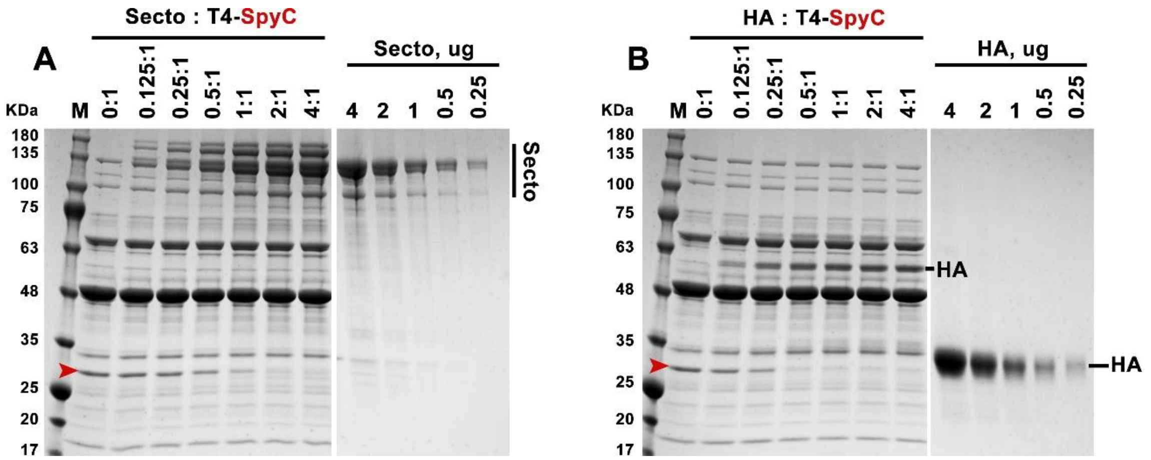

Figure S2. Optimization of S-ecto and HA-stem trimer display on T4 capsid. A) *In vitro* display of S-ecto trimers on T4-SpyC phage at increasing ratios of S-ecto trimer molecules to Soc binding sites (0:1 to 4:1). S-ecto standard was used for quantification. B) *In vitro* display of HA stem trimers on T4-SpyC phage at increasing ratios of HA stem trimer molecules to Soc binding sites (0:1 to 4:1). HA standard was used for quantification. Arrowheads depict the position of the Soc-SpyCatcher band which reduces in intensity as it gets conjugated with SpyTagged trimers with increasing SpyTagged trimers to Soc-SpyCatcher ratios.

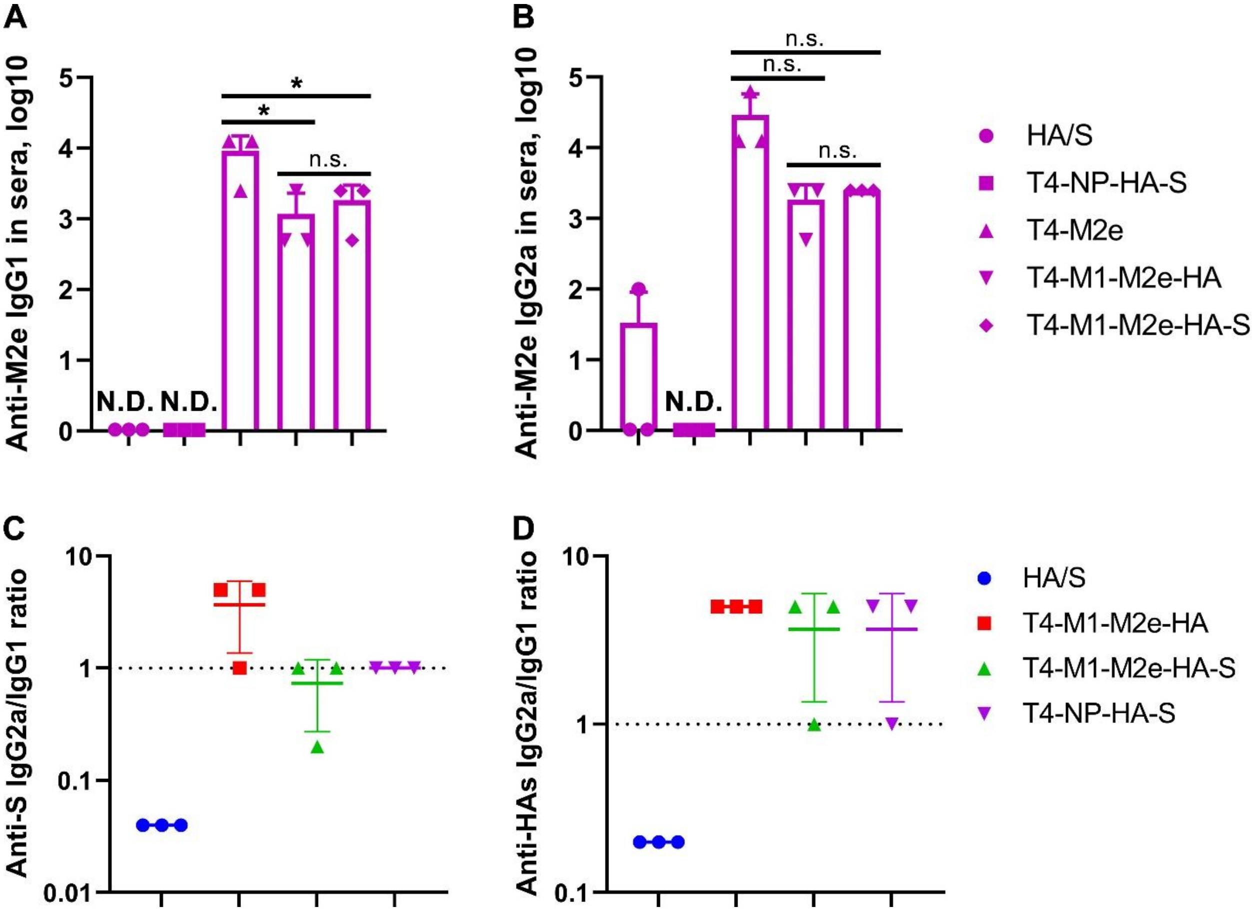

Figure S3. Intranasal immunization with T4-CoV-Flu vaccine elicited balanced systemic humoral immune responses. A) and B) Anti-M2e IgG1/IgG2a antibody responses in the sera of immunized BALB/c mice. C) and D) Comparison of IgG2a/IgG1 ratios for S-ecto-specific (C) and HA-stem-specific (D) antibodies in mouse sera across HA/S and various T4-CoV-Flu groups. The data are presented as means ± SD (n=3). Statistical comparisons among multiple groups were made using one-way analysis of variance (ANOVA) with Tukey's post *hoc* test. *, $P < 0.05$; n.s., not significant.

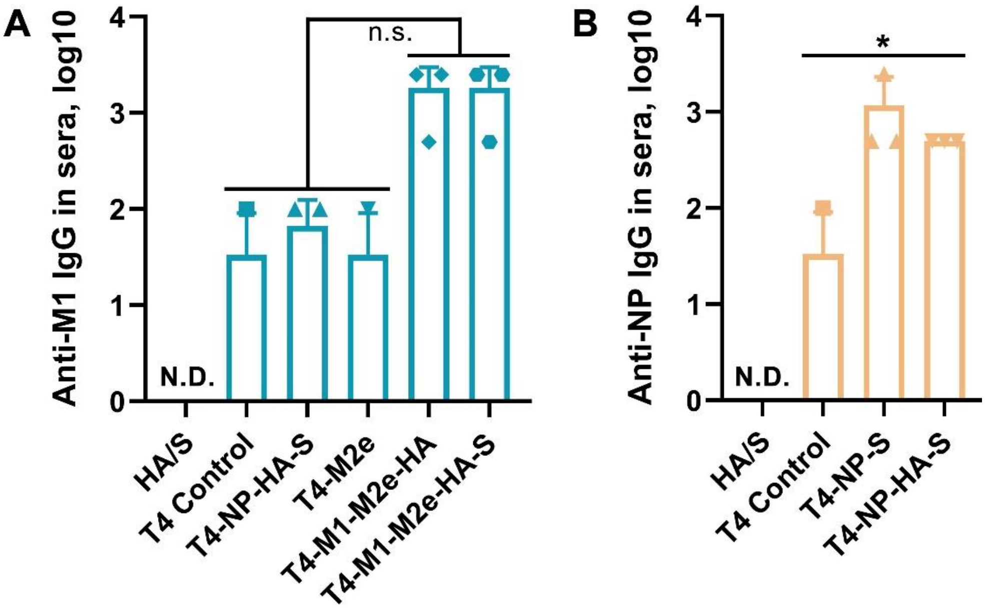

Figure S4. IgG antibody responses against M1 and NP in the sera of immunized mice. A) Anti-Flu M1 IgG in BALB/c mice sera. B) Anti-CoV NP IgG in ACE2 transgenic mice sera. ELISA was used to measure the reciprocal endpoint antibody titers. Data represent means ± SD. N.D. indicates not detected. The data are from three pooled independent experiments (n=5). Statistical comparisons among multiple groups were made using one-way ANOVA with Tukey's post *hoc* test. *, $P < 0.05$; n.s., not significant.

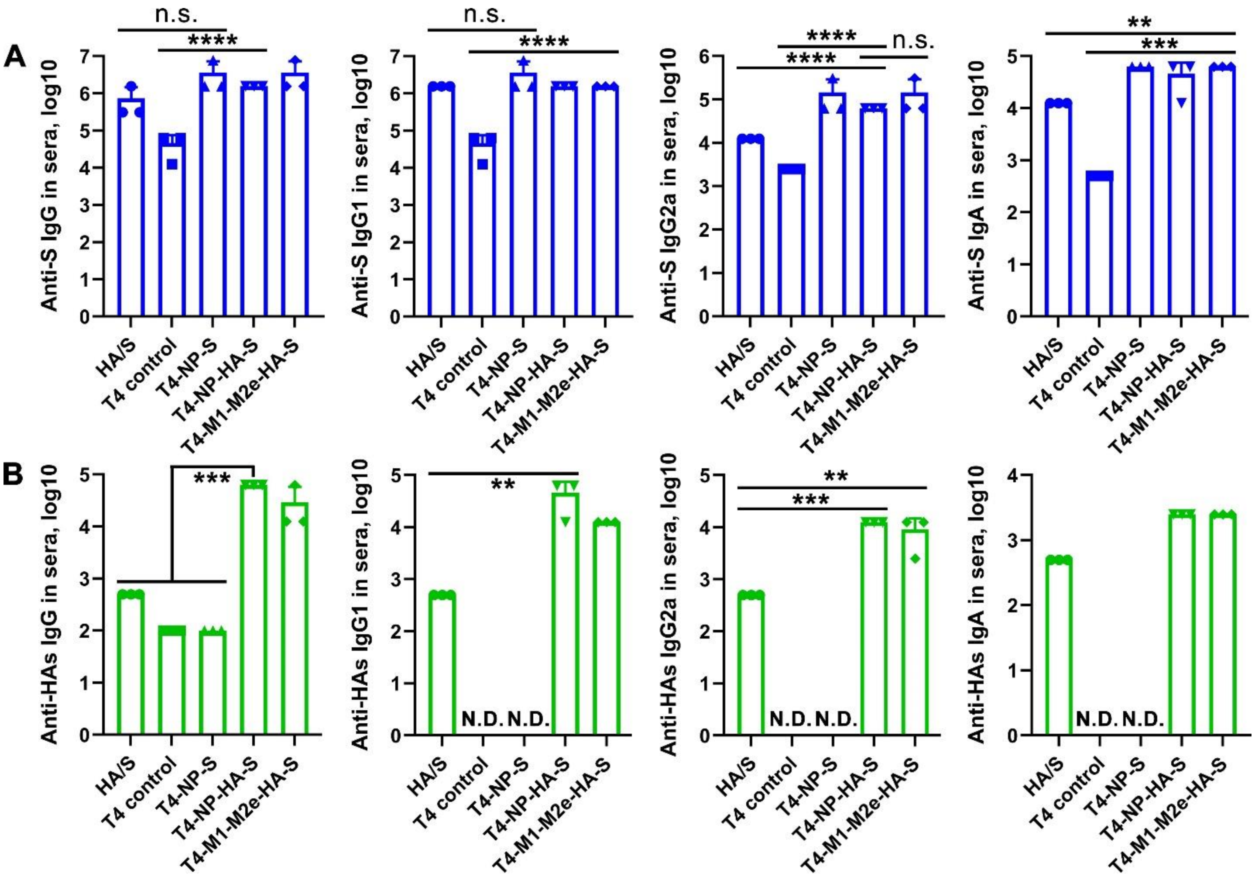

Figure S5. Antibody responses against CoV S-ecto and influenza HA-stem antigens in the sera of immunized hACE2 transgenic mice. ELISA was used to measure the reciprocal endpoint antibody titers for anti-S-ecto IgG/IgG1/IgG2a/IgA (A) and anti-HA-stem IgG/IgG1/IgG2a/IgA (B). Data represent means ± SD. N.D. indicates not detected. The data are from three pooled independent experiments (n=5). Statistical comparisons among multiple groups were made using one-way ANOVA with Tukey's post *hoc* test. **, $P < 0.01$; ***, $P < 0.001$; ****, $P < 0.0001$; n.s., not significant.

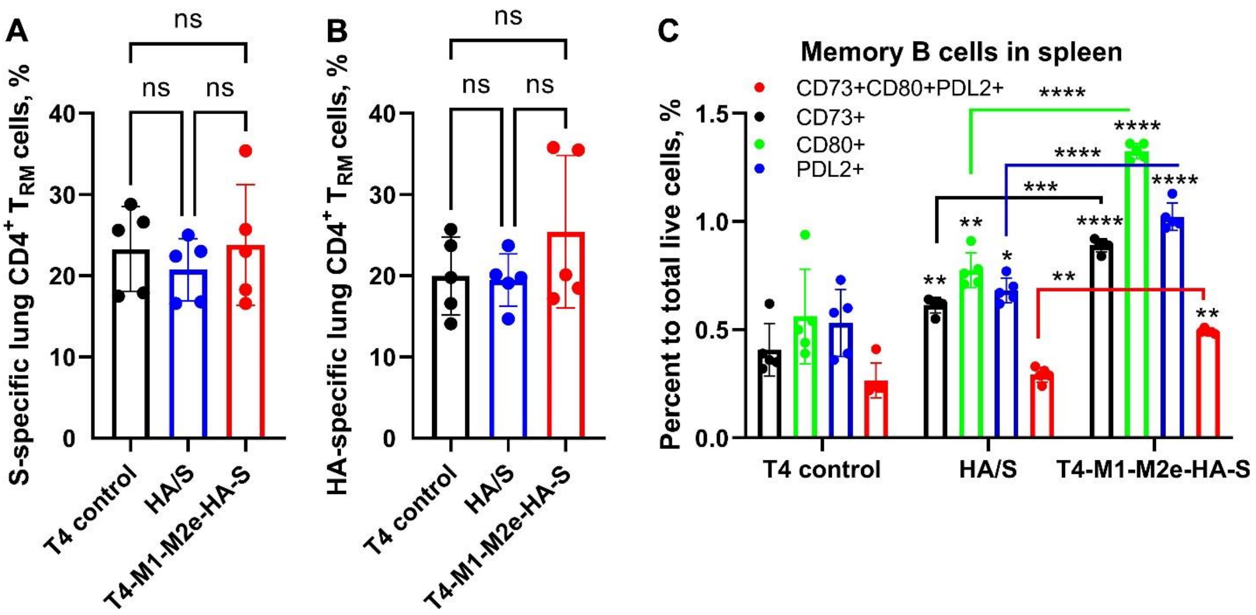

Figure S6. Comparison of memory B cells in spleen and CD4$^+$ T_{RM} Cells in the lung among T4 control, soluble HA/S trimer, and T4-M1-M2e-HA-S groups. A) and B) The percentages of S-ecto-specific CD4$^+$ T_{RM} (A) and HA-specific CD4$^+$ T_{RM} (B) in the lung. C) The percentages of memory B cells among total live cells in the splenocytes. Data represent means ± SD (n=5). One-way ANOVA with Tukey's multiple comparisons test was used to analyze statistical significance in (A and B). Statistical comparisons among multiple groups were made using two-way ANOVA with Tukey's post *hoc* test in (C). *, $P < 0.05$; **, $P < 0.01$; ***, $P < 0.001$; ****, $P < 0.0001$; ns, not significant.

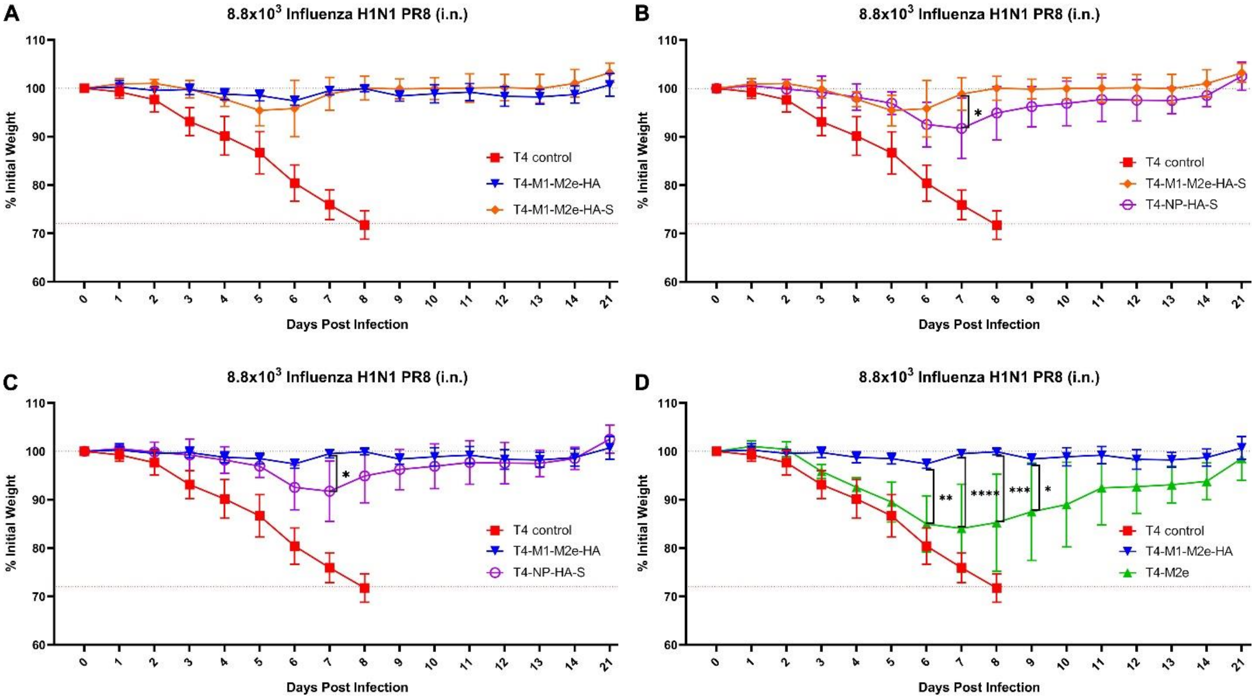

Figure S7. Body weight changes in T4-CoV-Flu intranasally-immunized mice following challenge with 8.8 x 10^3 Influenza H1N1 PR8. Comparisons of body weights are shown: A) Among T4 control, T4-MA-M2e-HA, and T4-M1-M2e-HA-S groups. B) Among T4 control, T4-M1-M2e-HA-S, and T4-NP-HA-S groups. C) Among T4 control, T4-M1-M2e-HA, and T4-NP-HA-S groups. D) Among T4 control, T4-M1-M2e-HA, and T4-M2e groups. Data represent means ± SD (n=5). Statistical comparisons among multiple groups were made using two-way ANOVA with Tukey's post *hoc* test. *, $P < 0.05$; **, $P < 0.01$; ***, $P < 0.001$; ****, $P < 0.0001$.

Antibodies used for memory B cells				
**Markers**				
**CD19**	FITC	6D5	BioLegend	115506
**CD3**	Alexa Fluor® 700	17A2	BioLegend	100216
**CD138**	BV650	281-2	Beckton Dickenson	564068
**CD38**	PE-Cy7	90	BioLegend	102718
**GL7**	Percp-cy5.5	GL7	BioLegend	144610
**IgD**	BV510	11-26c.a2	BioLegend	405723
**CD80**	BV421	16-10A1	BioLegend	104726
**CD73**	BV605	TY/11.8	BioLegend	127215
**PDL2**	PE	TY25	BioLegend	107206
**Antibodies used for T cell surface staining**				
**CD3**	Alexa Fluor® 700	17A2	BioLegend	100216
**CD4**	Brilliant Violet 785	GK1.5	BioLegend	100453
**CD8**	FITC	53-5.8	BioLegend	140403
**CD44**	BV510	IM7	BioLegend	103044
**CD62L**	BV711	MEL-14	BioLegend	104445
**CD127**	PE	S18006K	BioLegend	158204
**CD69**	BUV737	H1.2F3	BD Biosciences	612793
**CD103**	BUV395	M290	BD Biosciences	740238
**CD49a**	BV750	Hα31/8	BD Biosciences	746854
**CD11a**	BUV805	2D7	BD Biosciences	741919
**Antibodies used for T cell intracellular staining**				
**IFN-γ**	PerCP/Cyanine5.5	XMG1.2	Biolegend	505822
**IL-2**	PE-CF594	JES6-5H4	BD Biosciences	562483
**TNF-α**	eFluor 450	MP6-XT22	Thermo Fisher	48-7321-82
**IL-4**	BV650	11B11	BD Biosciences	564004
**IL-5**	APC	TRFK5	BD Biosciences	554396
**IL-17A**	PE/Cyanine7	TC11-18H10.1	Biolegend	506922

Table S1. Antibodies used for Flowcytometry
